# Determining population structure and hybridization for two iris species

**DOI:** 10.1002/ece3.964

**Published:** 2014-02-17

**Authors:** Jennafer A P Hamlin, Michael L Arnold

**Affiliations:** Department of Genetics, University of Georgia120 East Green St, Davison Life Sciences Building, Athens, Georgia

**Keywords:** Genotyping-by-sequencing, introgression, population genetics, southeastern US, species tree

## Abstract

Identifying processes that promote or limit gene flow can help define the ecological and evolutionary history of a species. Furthermore, defining those factors that make up “species boundaries” can provide a definition of the independent evolutionary trajectories of related taxa. For many species, the historic processes that account for their distribution of genetic variation remain unresolved. In this study, we examine the geographic distribution of genetic diversity for two species of Louisiana Irises, *Iris brevicaulis* and *Iris fulva*. Specifically, we asked how populations are structured and if population structure coincides with potential barriers to gene flow. We also asked whether there is evidence of hybridization between these two species outside Louisiana hybrid zones. We used a genotyping-by-sequencing approach and sampled a large number of single nucleotide polymorphisms across these species' genomes. Two different population assignment methods were used to resolve population structure in *I. brevicaulis*; however, there was considerably less population structure in *I. fulva*. We used a species tree approach to infer phylogenies both within and between populations and species. For *I. brevicaulis,* the geography of the collection locality was reflected in the phylogeny. The *I. fulva* phylogeny reflected much less structure than detected for *I. brevicaulis*. Lastly, combining both species into a phylogenetic analysis resolved two of six populations of *I. brevicaulis* that shared alleles with *I. fulva*. Taken together, our results suggest major differences in the level and pattern of connectivity among populations of these two Louisiana Iris species.

## Introduction

Evolutionary model systems abound, each holding their own promises and pitfalls dependent on the research regime, but many of these systems lack information regarding patterns of genetic diversity within natural populations beyond the reporting of estimates based on a handful of molecular markers. With current advances in genomic approaches, examining such geographic patterns of genetic variation has become possible (e.g., see June 2013 *Molecular Ecology* Special Issue: Genotyping By Sequencing in Ecological and Conservation Genomics) (Rieseberg 2013). Understanding these patterns is important because of the influence past geological events and genetic drift play on the demography and genetics of populations and species (Hewitt 2000). For example, some species may be continuously distributed throughout their range; however; many species are not found uniformly throughout their range and thus include small, isolated populations (Ellstrand and Elam 1993). Furthermore, evolutionary processes, such as natural selection and gene flow, can be reflected in the geographic sorting of genetic variation in populations (Slatkin 1987).

Gene flow can play an influential role in the evolutionary trajectories of populations through its ability to increase genetic diversity and change allele frequencies. In this investigation, we are defining populations as a group of individuals of the same species living in close enough proximity that any member of the group can potentially mate with any other member (Waples and Gaggiotti 2006). Our study also allows us to identify two different kinds of genetic diversity: (1) genetic diversity harbored within species due to population structure and (2) that which is shared among species due to hybridization. Population structure reflects shared alleles between individuals. Factors such as physical barriers, historic processes, or even variation in life histories can shape population differentiation thus resulting in variation in genetic connectivity among populations (Balloux and Lugon-Moulin 2002; Lowe and Allendorf 2010). Moreover, when gene flow occurs among populations, allele frequencies can become homogenized (Slatkin 1985). Reduced levels of gene flow and ecological differences associated with particular habitat patches can lead to local adaptation and may promote speciation (Barton and Hewitt 1985). In contrast, gene flow may generate new polymorphisms within a population and increase local effective population size, thereby opposing genetic drift (Wright 1931; Slatkin 1985).

Hybridization, as a result of gene flow between divergent but closely related taxa, can occur when species are found in sympatry and reproductive isolation is incomplete (Arnold 2006). One consequence of hybridization is the production of novel combinations of parental genotypes in otherwise isolated genomes (Arnold 1992, 2006). This will result in some unfit hybrid offspring, but it may also promote the exchange of genetic material between species (introgression), particularly if these novel gene combinations provide a selective advantage (Arnold and Hodges 1995; Arnold 2006; Arnold and Martin 2010). There are many well-known systems for studying hybridization and introgression such as *Mus musculus* and the genus *Heliconius*.

Recent work using *Mus musculus* concluded that directional introgression had occurred from *M. m. musculus* into *M. m. domesticus* supporting the hypothesis that alleles from one lineage may be adaptive in a sister lineage as well (Staubach et al. 2012). Another potential outcome of novel adaptations arising from introgressive hybridization is the formation of hybrid species. In this regard, work in the genus *Heliconius* has shown that *Heliconius heurippa* is of hybrid origin. Specifically, adaptive trait introgression produced a novel wing pattern in the homoploid hybrid species and resulted in reproductive isolation between the hybrid species and its parental species, *H. melpomene* and *H. cydno* (Salazar et al. 2010). Given the extensive work within these species, information is still lacking on the population genetics across these species' ranges.

The Louisiana iris species complex has been developed into a model system for the study of evolutionary genetics. Interest in this clade began with the postulation of a large number of species within Louisiana based on morphology (Small and Alexander 1931). Subsequently, Viosca (1935) and Riley (1938) demonstrated that many of these ‘species’ were actually hybrids between three taxa, *Iris brevicaulis*,*I. fulva,* and *I. hexagona*. Over the subsequent 70+ years, the Louisiana irises have been used to address a variety of evolutionary hypotheses, such as the link between hybrid fitness, introgression and adaptation, and, in the case of *I. nelsonii*, homoploid hybrid speciation (Randolph 1966; Arnold 1993; Taylor et al. 2011, 2013). However, much of this work has been focused on populations from the state of Louisiana where all of these species occur sympatrically. Although there is some information concerning population genetic variation in *I. hexagona* (Meerow et al. 2011), the geographic distribution of genetic diversity for two of the species, *I. brevicaulis* and *I. fulva*, has not been studied.

**Figure 1:**
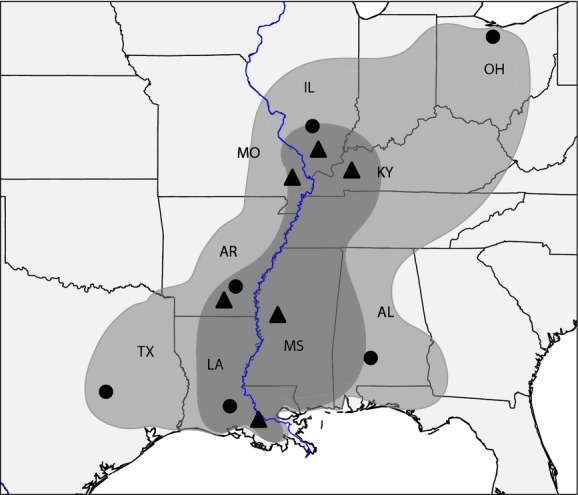
Range distribution maps for both *Iris brevicaulis* (light gray) and *Iris fulva* (dark gray). *Iris fulva* distribution is overlaid on *I. brevicaulis* distribution. Sympatric populations have only been found in Louisiana. Black dots are *Iris brevicaulis* and black triangles are *I. fulva* collection localities, respectively, from which individuals were sequenced. Abbreviations of states are shown, which corresponds to population names. The Mississippi River is outlined in blue.

*Iris brevicaulis* and *I. fulva* occur throughout the Southeastern United States with overlapping ranges along the Mississippi River and *I. fulva's* distribution nested within *I. brevicaulis* (Fig. 1). *Iris brevicaulis* is distributed as far north as Ohio and as far west as Texas. *Iris fulva* is geographically more restricted, being associated with the alluvial valley of the Mississippi River. The Lower Mississippi Alluvial Valley (LMAV), which stretches from Illinois to the Gulf of Mexico, is the historic floodplain of the lower Mississippi (Stanturf et al. 2000), and a number of common phylogeographic patterns have been reported across a wide range of codistributed taxa (Soltis et al. 2006). From the present study, we address the following questions regarding *I. brevicaulis* and *I. fulva*: (1) How is genetic diversity partitioned and how are populations structured? (2) Does population structure coincide with potential geographic barriers to gene flow, such as the Mississippi River? (3) Is there evidence of hybridization between these species beyond the hybrid zones defined in the state of Louisiana?

## Materials and Methods

### Study system and sampling

Both iris species occur in or near wetland systems, but each species differs with regard to microhabitat, floral morphology, and pollination system (Viosca 1935). *Iris fulva* is found at lower elevations in intermittently flooded, forested wetlands. *Iris brevicaulis* occurs at slightly higher elevation in mixed hardwoods (Viosca 1935; Cruzan and Arnold 1993; Johnston et al. 2001). *Iris fulva* flowers are a deep crimson color, have protruding anthers, lack nectar guides, and produce large volumes of nectar (Viosca 1935; Wesselingh and Arnold 2000). Hummingbirds and butterflies pollinate these flowers (Viosca 1935; Wesselingh and Arnold 2000; Martin et al. 2008). Floral characteristics of *I. brevicaulis* are typical of a bumblebee pollination syndrome; floral color varies from light blue to deep blue with marked nectar guides, stiff upright sepals, and strong scent. However, even given these floral differences, *I. brevicaulis* and *I. fulva* are known to form hybrid zones within the state of Louisiana (Viosca 1935; Foster 1937; Riley 1938). Additionally, individuals found in hybrid zones are sometimes characterized by distinct phenotypes and genotypes that are associated with particular ecological traits (Cruzan and Arnold 1994).

During the summer of 2011 and 2012, we performed collections for both species. These collections included populations throughout the species ranges (Fig. 1). For *I. brevicaulis*, collections occurred from eight states resulting in a total of 15 localities. For *I. fulva*, collections occurred in seven states resulting in a total of ten collection localities. A subset of the populations collected for both species was used in this study (Table 1). Sequenced populations included samples from both sides of the Mississippi River. We did not sequence individuals found in previously documented hybrid zones.

**Table 1:** Collection information for (a) *Iris brevicaulis* populations; (b) *Iris fulva* populations.

State	Id	Longitude	Latitude	*n*
(a)				
Alabama	AL	−86.239	31.920	8
Arkansas	AR	−91.499	34.854	8
Illinois	IL	−89.113	38.926	8
Louisiana	LA	−92.051	30.519	8
Ohio	OH	−82.551	41.361	8
Texas	TX	−96.201	30.568	8
(b)				
Arkansas	AR	−91.952	34.235	8
Illinois	IL	−89.396	37.443	8
Kentucky	KY	−89.274	36.539	8
Louisiana	LA	−90.819	29.877	7
Mississippi	MS	−90.910	33.791	8
Missouri	MO	−90.196	36.973	8

### DNA extraction, library construction and sequencing

DNA was extracted from eight individuals for six populations of each species throughout the species' range (Fig. 1). Extractions were performed using the Qiagen DNeasy plant kit (Qiagen, Valencia, CA). Extracted DNA was sent to the Cornell Institute for Genomic Diversity for genotyping-by-sequencing (GBS) (Elshire et al. 2011). GBS is similar to RAD sequencing in that it generates reduced representation libraries by digesting DNA with a restriction enzyme; however, GBS differs from RAD sequencing in that adaptor ligation results in reduced sample handling and DNA fragments are not size selected (Elshire et al. 2011). Libraries were prepared from 48 *I. brevicaulis* individuals, 47 *I. fulva* individuals, and one blank (i.e., control) for sequencing. DNA from each individual was separately digested using *Eco*T221, a six base cutter, and the fragmented DNA was then ligated to a barcoded adaptor and a common adaptor. Within a 96-well plate, each well contained DNA from a different individual and a barcode adaptor unique to that well. Individuals were barcoded to allow discovery of genetic variation both within and between species. The resulting libraries were sent for sequencing using single-end 100-bp reads on the Illumina HiSeq 2000 with 48 samples sequenced per lane.

### SNP discovery, genotyping, and summary statistics

The nonreference pipeline, Universal Network-Enables Analysis Kit (UNEAK; http://www.maizegenetics.net/gbs-bioinformatics), was used for SNP discovery and genotyping both between and within species. UNEAK takes raw Illumina sequence files and converts them into individual genotypes. Reads are retained, and trimmed to 64 bp, when they possess a barcode, cut site, and no ‘N's in the first 64 bp of sequence after the barcode. Identical reads are clustered into tags and counts of these tags present in each barcoded individual are stored. Pairwise alignment, then, identifies tag pairs having a single base pair mismatch, and these single base pair mismatches are considered candidate SNPs. Any tag pair that contains more than one mismatch is discarded to minimize SNPs resulting from alignment of paralogous sequences. Error tolerance rate was set at 0.03, so that only reciprocal pairs of tags are retained for SNP calling according to standard protocols of Cornell Institute for Genomic Diversity.

Subsequent filtering of tags was carried out, using the program TASSEL 4.0 in order to identify SNPs, both within and between species (Bradbury et al. 2007). A few individuals were not used in identifying SNPs, because those samples failed during sequencing. After removing failed samples and setting a threshold of 500k reads, 20% missing data (‘N's), and a minor allele frequency of >1% (or the minimum frequency at which a common allele must occur), a total of 67 individuals were used to generate a filtered set of SNPs between species. The total number of individuals used to call SNPs within a species was 43, which we excluded the failed samples and set a threshold of 20% missing data (‘N's), and a minor allele frequency of >1%. Pairwise *F*_ST_ values, observed and expected heterozygosity, inbreeding coefficient (*G*_IS_), which is analogous to *F*_IS_ by Wright (Wright 1951), and isolation-by-distance (IBD) were estimated for each SNP, and their average across loci was computed using Genodive (Meirmans and Van Tienderen 2004).

### Population assignments

Individual and population assignments were conducted using the program STRUCTURE version 2.3.4, both within and between species (Pritchard et al. 2000). For all analyses, we used a model of admixture to determine the number of population clusters (K) with a burn-in of 1,000,000 and 10,000,000 iterations. Analyses were repeated 10 times for each k value, ranging from 1 to 7 within species or 1 to 13 between species. The average and standard deviation (SD) of the natural log probability of each model were used to calculate ΔK (Evanno et al. 2005) using Structure Harvester (Earl and vonHoldt 2012). The program clumpp, set at the default settings, was used to assess the similarity between replicate STRUCTURE results (Jakobsson and Rosenberg 2007).

Discriminant analysis of principle components (DAPCs) (Jombart et al. 2010) from the package adegenet (Jombart 2008) version 1.2.8 in R (2009) were also used to detect the number of genetic clusters. This technique uses a non-model-based multivariate approach. DAPC first transforms genetic data into uncorrelated components using principle component analysis (PCA) and then performs a discriminant analysis on the retained principle components (PCs). We used the find.clusters function of DAPC to infer the most likely number of clusters. To calculate the probability of assignment of individuals to each of these clusters using DAPC, we determined the optimal number of principle components; here, the optimal number of PCs retained was N/3; *N* = number of samples as advised in the manual. We calculated the Bayesian Information Criterion (BIC) for *K* = 1–7, where *K* = number of populations. Optimal number of populations was identified as the one in which the BIC was the lowest value and after which the BIC either increased or decreased by the least amount.

### Phylogenetic analyses

Maximum-likelihood phylogenies were inferred using RAxML (Stamatakis 2006). For each individual, SNPs were concatenated into one sequence. RAxML was run using a GTR+G model of nucleotide substitution. The −D option, which stops the ML searches when they have reached the asymptotic convergence phase, was used. The criterion for stopping the searches is based on computing the Robinson-Foulds (RF) distance (Robinson and Foulds 1981) between two consecutive intermediate trees. If the RF distance between two consecutive trees is smaller than 1%, the ML search is stopped. Support for nodes in the RAxML inferred tree was assessed using a bootstrap analysis with 100 replicates. However, treating SNPs as a contiguous sequence violates assumptions about recombination, and because of this, we also inferred a species tree.

Species trees from SNPs were inferred using SNAPP (SNP and AFLP Package for Phylogenetic analysis) with the default settings. SNAPP, part of BEAST 2.0, is a package for inferring species trees and species demographics from independent (unlinked) biallelic markers (Bryant et al. 2012). This package implements a full coalescent model to integrate over all possible gene trees rather than sampling them explicitly. SNAPP was run for at least 10,000,000 generations with sampling every 1000 generations. Convergence of parameters onto posterior distribution was assessed using Tracer version 1.5 (http://tree.bio.ed.ac.uk/software/tracer/). All parameters had effective sample size (ESS) values > 250. Convergence onto posterior topology was assessed using AWTY (Nylander et al. 2008). All samples before convergence were removed as burn-in, and maximum clade credibility trees were generated using TreeAnnotator within the BEAST 2.0 package. Finally, due to lack of an outgroup, all trees are midpoint rooted.

## Results

### Genotyping and population statistics

A total of 151,189 SNPs were identified from individuals from all populations of *I. brevicaulis*. After filtering out failed samples, missing data ≤20% and a minimum allele frequency of >1%, 387 SNPs were retained for 43 individuals. For *I. fulva*, 97,432 SNPs were called from individuals from all populations. Applying the same filtering as above, a total of 560 SNPs were retained for analyses from 43 individuals. There were 196,133 SNPs in total generated when *I. brevicaulis* and *I. fulva* were run through the UNEAK pipeline. For 67 individuals combined, and the same filtering protocol except with a minimum of 500k reads, a total of 1140 SNPs were retained. To determine whether the tag pairs were derived from nuclear or chloroplast DNA, a subset of the tag pairs (*n* = 200) were blasted against the nonredundant, nucleotide database collection using BLASTN. Almost all tag pairs examined resulted in E-value scores which were not lower than 10^−4^; however, four tag pairs had an E-value of below 10^−4^ that matched sequences from chloroplast DNA. One tag pair with a significant E-value was associated with an *I. brevicaulis* IRRE transposon marker.

For *I. brevicaulis,* within-population observed heterozygosity (*H*_o_) ranged from 0.101 to 0.259 and expected frequency of heterozygotes over all populations (*H*_e_) ranged from 0.144 to 0.217. The inbreeding coefficient (*G*_IS_) ranged from 0 to 0.3, with three population *G*_IS_ values significantly different than zero (IB_AL, IB_LA, and IB_OH) (Table S1a). For *I. fulva*, within-population observed heterozygosity (*H*_o_) ranged from 0.314 to 0.561 and expected frequency of heterozygotes over all populations (*H*_e_) ranged from 0.357 to 0.403. The inbreeding coefficient (*G*_IS_), for *I. fulva*, was approximately zero for all populations (Table S1b). Genetic differentiation (pairwise population *F*_ST_) for *I. brevicaulis* populations was moderately high (Table 2a). In contrast for *I. fulva,* the degree to which populations were different from one another was much lower (Table 2b).

**Table 2:** Pairwise *F*_ST_ values for (a) *Iris brevicaulis* populations; (b) *Iris fulva* populations.

	IB_AL	IB_AR	IB_IL	IB_LA	IB_OH
(a)					
IB_AL	–				
IB_AR	0.53	–			
IB_IL	0.56	0.19	–		
IB_LA	0.2	0.51	0.54	–	
IB_OH	0.57	0.25	0.15	0.55	–
IB_TX	0.56	0.27	0.35	0.52	0.38

	IF_AR	IF_IL	IF_KY	IF_LA	IF_MS
(b)					
IF_AR	–				
IF_IL	0.04	–			
IF_KY	0.06	0.01	–		
IF_LA	0.04	0.05	0.06	–	
IF_MS	0.05	0.03	0.06	0.04	–
IF_MO	0.03	0.02	0.04	0.03	0.02

Here, abbreviations are based on the state where populations are collected. AL, Alabama; AR, Arkansas; IL, Illinois; KY, Kentucky; LA, Louisiana; MS, Mississippi; MO, Missouri; OH, Ohio; TX, Texas.

No isolation-by-distance was detected (data not shown) in either species. The slope of the regression line in the Mantel test (correlation of geographic and genetic distance) was not significantly different than zero (*I. brevicaulis*:*R*^2^ = 0.051, *P* = 0.192; and *I. fulva*;*R*^2^ = 0.118, *P* = 0.130).

### Population assignments

**Figure 2:**
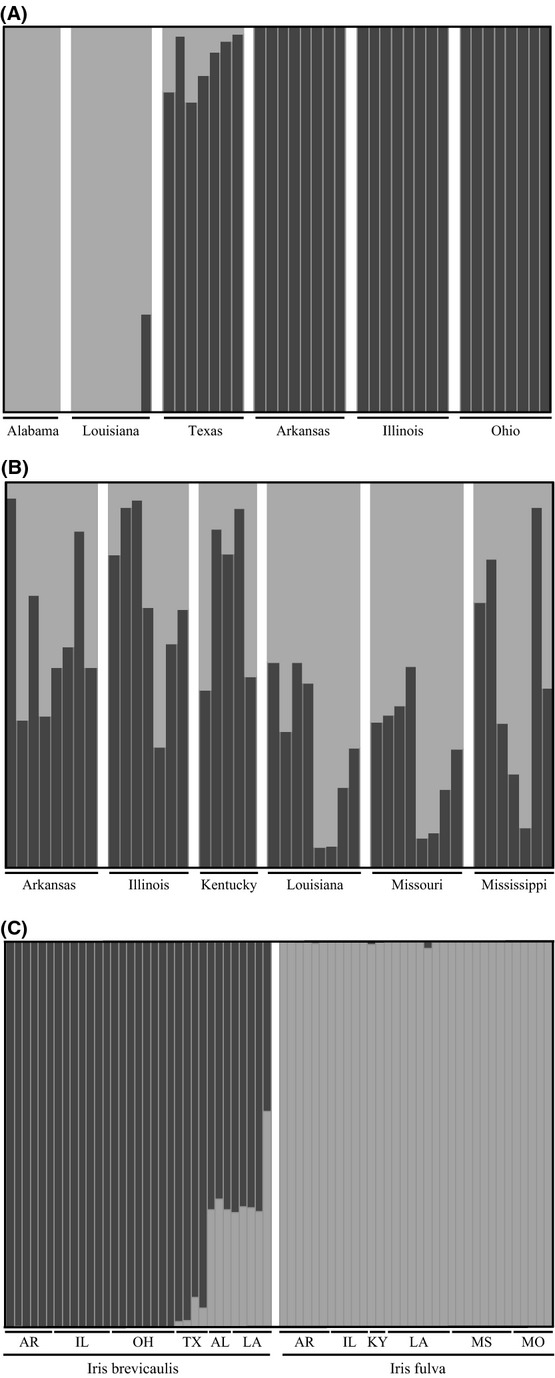
Plots of posterior probabilities of assignment of each individual into its associated cluster based on the STRUCTURE analysis. The results are grouped by collection localities for each individual. Each vertical bar represents a different individual from one of twelve populations. (A) *Iris brevicaulis* plot of posterior probabilities of group assignments where *K* = 2. (B) *Iris fulva* plot of posterior probabilities of group assignment where *K* = 2. (C) *Iris brevicaulis* and *I. fulva* species plot of posterior probabilities of group assignments generated where *K* = 2.

We analyzed models (using STRUCTURE) for within a species with 1–7 clusters and between species with 1–13 clusters. ΔK, the statistic used to detect the most likely number of clusters, *K*, peaks at *K* = 2 for *I. brevicaulis* (Fig. 2A). *Iris brevicaulis* individuals from Alabama and Louisiana were placed in one cluster along with Texas, to a lesser extent while all other individuals formed a second cluster. For *I. fulva,* ΔK peaks at both *K* = 2 (Fig. 2B) and *K* = 4 (Figure S1), although with only a slight difference between the two. At *K* = 2, we do not see two distinct clusters, but a proportion of each individual's genome is shared with both clusters. Because the proportion of the individuals assigned to each of the two populations is roughly symmetric, we conclude that there is basically no population structure. When individuals from both species were combined and analyzed using STRUCTURE, the optimal number of clusters is *K* = 2 (Fig. 2C). Individuals within the two distinct clusters were associated with previously assumed species but with *I. brevicaulis* individuals from Alabama, Louisiana, and Texas, though to a lesser extent, clustering with *I. fulva*.

**Figure 3:**
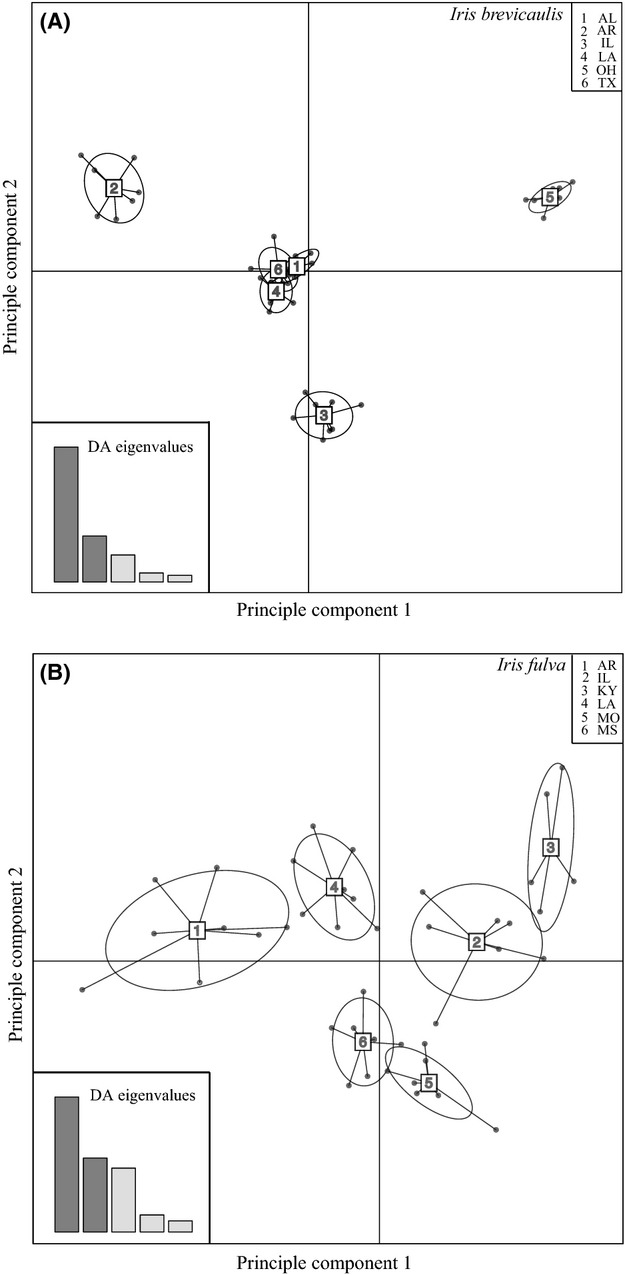
Samples are assigned to their genetic cluster by discriminant analysis of principle components (PCs). The bar graph inset displays the eigenvalues of the five principal components in relative magnitude and illustrates the variation explained by the five PCs. The 67% inertia ellipses are drawn for each cluster representing the variance of both PCs. (A) Principle component scatter plot for *Iris brevicaulis*. (B) Principle component scatter plot for *I. fulva*.

For *I. brevicaulis* DAPC analysis, the BIC gave the most support to three genetic clusters (Figure S2A), which differs from STRUCTURE (Fig. 2A). The first principle component separated populations 2 and 5 (Arkansas and Ohio) from 1, 3, 4, and 6. The second component separated these last four populations into two groups, southern populations that are closest to the hybrid zone (clusters 1, 4, and 6; Alabama, Louisiana, and Texas, respectively) and a northern population (cluster 3; Illinois) (Fig. 3A). In the *I. fulva* DAPC analysis, the BIC gave the most support for one genetic cluster (Figure S2B). However, there is subtle genetic structure where the first principle component separated populations 1, 4, and 6 from populations 2, 3, and 5. The second component separated the first group into populations 1 and 4 (Arkansas and Louisiana) from population 6 (Mississippi). For the second group (2, 3, and 5), the second function separated 2 and 3 (Illinois and Kentucky) from 5 (Missouri) (Fig. 3B).

### Phylogenetic analysis

Nodes with <50% support were collapsed for RAxML trees. Most nodes in the *I. fulva* tree were collapsed resulting in a polytomy. The *I. brevicaulis* phylogeny generated by RAxML showed some structuring with Alabama and Louisiana genotypes forming one clade (bootstrap support = 98%), which was sister to all other samples (Figure S3). The remainder of the genotypes demonstrated a geographic partitioning with populations becoming more nested with increasing latitude. When species were combined, individuals from Alabama and Louisiana formed one clade (bootstrap support = 98%) that was more closely related to *I. fulva* genotypes than to other *I. brevicaulis* individuals or populations (Figure S4).

**Figure 4:**
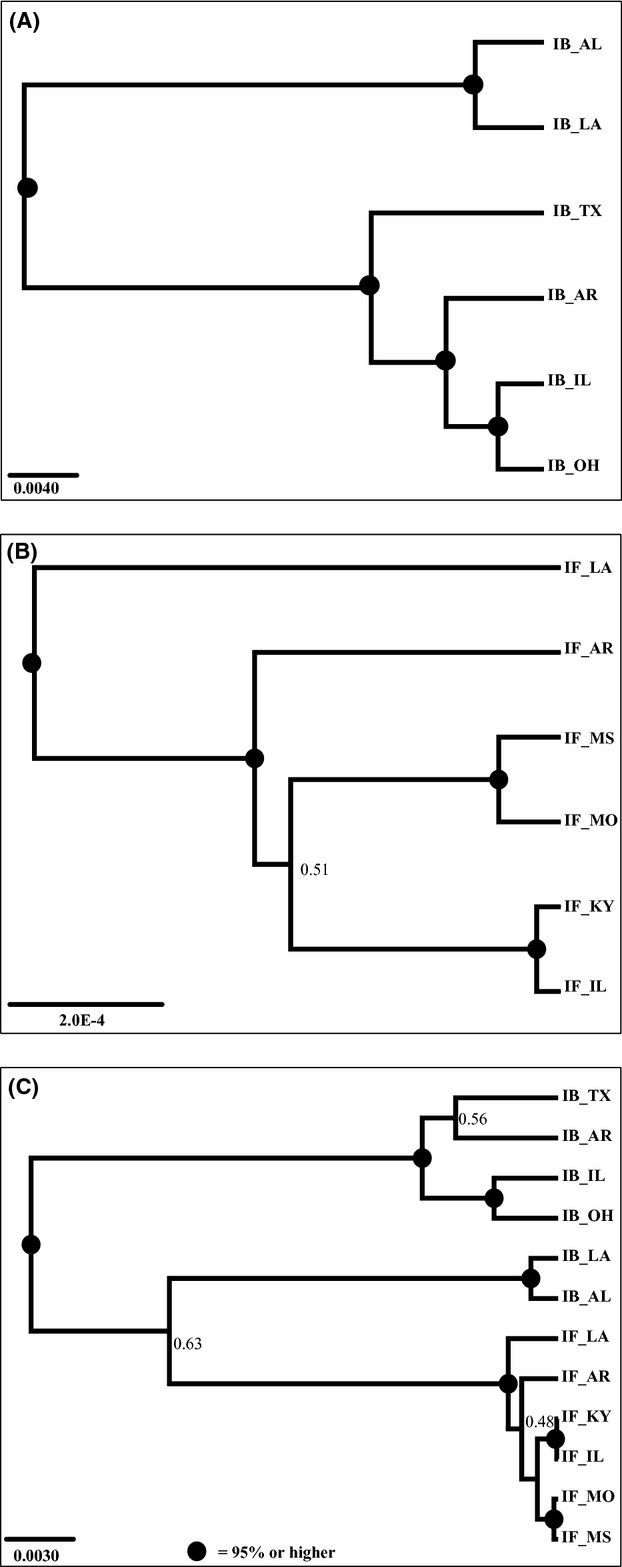
Species tree inferred using SNAPP with independent biallelic markers. Proportion of posterior support given at each node and nodes with support = 95% or higher have no label, but have a black circle (A) *Iris brevicaulis* populations (B) *Iris fulva* populations (C) *I. fulva* and *I. brevicaulis* populations.

SNAPP species trees were constructed by defining populations as different species. We detected high posterior support for each of the nodes ranging from 0.982 to 1 for *I. brevicaulis* (Fig. 4A). Population level clades were similar to the phylogeny estimated using RAxML (Figure S4). There was also high support for Alabama and Louisiana being sister to each other. The *I. fulva* species tree reflected population differentiation with high posterior support for (1) the Louisiana sample being sister to all other populations and (2) the Arkansas sample being sister to the four other populations (Fig. 4B). For the remainder of the populations (Illinois, Kentucky, Missouri, Mississippi), the posterior support was much lower (i.e. 0.513). When samples of both species were combined for the species tree analysis, the *I. brevicaulis* Alabama and Louisiana populations were again more closely related to *I. fulva*, than to other *I. brevicaulis* samples (Fig. 4C).

## Discussion

Understanding how individuals, gametes, and genes move between populations and species is fundamental to studies of ecology and evolution. For example, habitat fragmentation can lead to reduced population size and gene flow with resulting decreases in individual heterozygosity and local genetic diversity over time (Ellstrand and Elam 1993). This can result in inbreeding depression and/or differentiation at a regional or range-wide scale (Ellstrand and Elam 1993). Specific biotic regions, such as the Southeastern United States, provide an ideal backdrop to test hypotheses regarding the factors that may have affected genetic partitioning within species (Soltis et al. 2006).

The present study used a genotyping-by-sequencing (GBS) approach to genotype individuals. Advantages of using a GBS approach include genotyping nonmodel, large genome, organisms using a large panel of SNPs at a cost that is comparable to traditional Sanger sequencing for a handful of molecular markers. Library preparation is much simpler than traditional RAD approaches and can be performed relatively quickly and is amenable to a high level of multiplexing. Essentially, GBS does marker discovery and genotyping simultaneously compared with independent marker discovery, assay designs, and genotyping following the classical approach. Lastly, by generating fragments of DNA spread throughout the genome, one can potentially sequence regions inaccessible to other sequencing approaches. However, GBS suffers the same bioinformatics hurdles as all next-generation sequence methods such as generating and analyzing massive amounts of data, dealing with complex genomes, and the presence of missing data.

### Population structure and the Mississippi River

While the Mississippi River has been documented to be a barrier to gene flow in a number of species (Soltis et al. 2006), this was not found to be the case for *I. brevicaulis* and *I. fulva*. Our results are in line with those previously reported for two species of frog, *Rana catesbiana* and *Pseudacris crucifer* (Austin et al. 2004). These two species showed geographic overlap of genetic diversity suggesting that this waterway has not been a major barrier to gene flow (Austin et al. 2004). In contrast, one species, the Loblolly pine (*Pinus taeda*) does show differentiation between populations located on either side of the Mississippi River, consistent with the river being a barrier to gene flow (Al-Rabab'ah and Williams 2002; Eckert et al. 2010). It was hypothesized that differentiation among the pine populations may have resulted from separate Pleistocene refugia on either side of the river (Al-Rabab'ah and Williams 2002).

In contrast to the Loblolly pine, *I. brevicaulis* does not appear to have been influenced by the Mississippi River acting as a barrier to gene flow. Thus, we did not find populations on either side of this waterway to be more similar to one another than those located on the opposite side. Instead, it appears that populations closest to the Louisiana hybrid zones form one separate genetic unit relative to other samples. This was evident from the Bayesian clustering method, STRUCTURE, as well as the DAPC analysis. *Iris brevicaulis* also demonstrated high levels of genetic differentiation or pairwise *F*_ST_ values. Alabama, Louisiana, and Texas populations have the highest levels of differentiation calculated by *F*_ST_ and lowest levels of genetic diversity (heterozygosity) compared with the remaining populations. Additionally, both the RAxML and SNAPP trees failed to detect genetic differentiation due to the Mississippi River. Specifically, we did not resolve two reciprocally monophyletic groups of populations distributed on opposite sides of the Mississippi River. Instead, the phylogeographic pattern for *I. brevicaulis* is one of higher latitude populations being more derived relative to more southerly distributed samples.

Based on the geographic distribution of *I. brevicaulis*, it is possible to test whether this species conforms to the central–marginal hypothesis (Eckert et al. 2008). The central–marginal hypothesis states that peripheral populations exhibit low genetic diversity and greater genetic differentiation compared with central populations. Additionally, effective population size and the rate of migration should be highest at the range center than at range margins; this results from smaller effective population size and greater geographic isolation of the range margins. Some *I. brevicaulis* populations (i.e., Alabama or Arkansas) would be expected to exhibit characteristics typical of central populations, such as having high levels of genetic diversity as measured by expected heterozygosity (Eckert et al. 2008). The *I. brevicaulis* Arkansas population does indeed reflect characteristics of central populations. In contrast, we detected the opposite pattern for the *I. brevicaulis* sample from Alabama, possibly resulting from high levels of inbreeding within this latter population.

In contrast to *I. brevicaulis, I. fulva* showed no geographic clustering, as reflected by the estimated levels of shared genetic variation among all populations (Fig. 2A). Furthermore, there were low levels of genetic differentiation among populations as indicated by pairwise *F*_ST_ values. We were unable to resolve the *I. fulva* population phylogeny using a maximum-likelihood method, which required us to concatenate all SNPs (*n* = 560) into one single locus. We were, however, able to resolve the population phylogeny using a species tree approach. We found that the Louisiana population was a sister lineage to all other populations. Future analyses estimating migration rates between collection localities should provide further definition of patterns of gene flow among *I. fulva* populations.

Despite the fact that the two iris species studied here are closely related and have similar life histories, the patterns of genetic diversity suggest two different evolutionary histories. First, it appears that *I. brevicaulis* is experiencing limited gene flow between populations, while *I. fulva* is not. Our results are not consistent with a geographic barrier (i.e. the Mississippi River) to gene flow and there are other processes that could lead to the observed patterns of genetic differentiation. Within plants, selection on various floral morphologies has been well documented and shown to be influenced by both abiotic and biotic factors (Fenster et al. 2004; Strauss and Whittall 2006; Bomblies 2010). In animal pollinated species, patterns of pollen dispersal and receipt depend on the behavior and effectiveness of pollinators (Waser and Price 1983; Galen 1989; Campbell et al. 1996). Floral traits, such as color, fragrance, shape, and size, reflect various ‘pollinator syndromes’. *Iris brevicaulis* is primarily bumblebee pollinated (Viosca 1935; Bouck et al. 2007), and *Iris fulva* is predominately pollinated by either hummingbirds or butterflies (Viosca 1935; Bouck et al. 2007).

Fragmentation of habitats can affect bee populations and thereby disrupt plant–pollinator interactions (Steffan-Dewenter and Tscharntke 1999). For example, Steffan-Dewenter and Tscharntke (1999) found that increasing isolation of small habitat islands resulted in both decreased abundance and species richness of flower-visiting bees. From this, they concluded that habitat connectivity is essential to maintain not only abundant and diverse bee communities, but also plant–pollinator interactions in wild plants. In contrast, ruby-throated hummingbirds (*Archilochus colubris*) are well known for spring migratory flights that cover great distances encompassing most of eastern North America (Depamphilis and Wyatt 1989). This migratory pattern led to the conclusion that ruby-throated hummingbirds contributed to long-distance gene flow in buckeyes (Depamphilis and Wyatt 1989). The patterns of limited gene flow between *I. brevicaulis* populations versus higher rates of gene flow among geographically widely spaced *I. fulva* populations are thus consistent with the behavior of their major pollinators.

Additional to the possible role of pollinator systems in limiting and promoting gene flow within the two Louisiana iris species, their divergent ecological associations may also be affecting the other avenue of gene flow available for plant species, that of seed dispersal. Because iris seeds float, they can be dispersed via waterways. As *I. fulva* is closely associated with the LMAV, which is prone to flooding, seed dispersal may have led to the higher estimated levels of gene flow for this species. Additionally, whole parts of plants could be washed from one place to another via flooding, potentially spreading the plants clonally across the flood plane. For *I. brevicaulis*, it is more likely that there is a lack of connectivity from seed dispersal due to its greater separation from waterways (Viosca 1935; Cruzan and Arnold 1993).

### Evidence of hybridization beyond Louisiana hybrid zones

Outcomes from natural hybridization are varied and often have significant evolutionary consequences (Arnold 2006). In the present study, we first tested for introgression between the two iris species using STRUCTURE. This analysis resolved two distinct clusters consisting of either *I. brevicaulis* or *I. fulva* individuals. However, a proportion of the genome of Alabama, Louisiana, and Texas *I. brevicaulis* individuals was associated with the *I. fulva* cluster. A similar finding was apparent from the phylogeny containing both species, in which *I. brevicaulis* individuals from Alabama and Louisiana collection localities formed a monophyletic clade that was more closely related to *I. fulva* than to other *I. brevicaulis*, however node support was fairly low (0.63).

Previous analyses of the Louisiana irises found that, within a hybrid population, pollinator-mediated pollen movement between two species was more likely than seed movement. Specifically, some individuals were exclusively the products of pollen transfer from *I. fulva,* or hybrid plants, into a third species of the Louisiana iris species complex, *I. hexagona* (Arnold et al. 1991). However, it was inferred that both seed-and pollen-mediated gene flow occurred and/or that this was a region of historic overlap. Regarding the inferred introgression into the Alabama, Louisiana, and Texas *I. brevicaulis* populations, *I. fulva* has been previously recorded from these states. Though we failed to locate *I. fulva* in either Alabama or Texas, it is likely that past sympatry between these two species generated the patterns of admixture found in the *I. brevicaulis* populations.

Past analyses within the Louisiana irises have consistently detected asymmetrical introgressive hybridization. In regard to *I. fulva* and *I. brevicaulis*, this asymmetry was reflected in a higher frequency of transfer of alleles from the former into the latter species (Cruzan and Arnold 1994; Martin et al. 2005, 2006; Tang et al. 2010). Here, we demonstrate a pattern that is consistent with the previous findings. Thus, the population genetic variation detected here suggests a further case of asymmetric introgression between these two species—from *I. fulva* into the Alabama, Louisiana, and Texas populations of *I. brevicaulis*.

It has been known for decades that introgression is not homogeneous across the genome (e.g., Key 1968); the frequency of exchange of specific genomic regions between species depends on both demographic and deterministic processes (see Arnold and Martin 2010; for a review). In a number of documented cases of introgression, it has been shown that some loci move freely between genomes while others remain highly differentiated (Nosil et al. 2009). Loci that do not introgress are thought to be associated with genomic regions that contribute to reproductive isolation.

In a previous study of *I. fulva* and *I. brevicaulis,* Tang et al. (2010) found that alleles from *I. fulva* were significantly favored compared with regions from *I. brevicaulis*, but concluded that both species genomes were permeable to introgression. Additionally, using both greenhouse studies and natural field experiments, Martin et al. (2005, 2006) documented that adaptive trait introgression had occurred between *I. brevicaulis* and *I. fulva*. These authors detected an increased survival of genotypes of a backcross-1 generation toward *I. brevicaulis*, when these genotypes contained significantly more alleles from *I. fulva*. These patterns reflected signatures of positive selection for certain introgressed alleles (i.e., adaptive trait introgression), which apparently underlie adaptations to ecological settings within these iris species (Martin et al. 2005, 2006). Further examination of the loci used in this study will elucidate whether those loci are adaptive.

Adaptive trait introgression has been inferred in a number of organisms other than iris. For example in *Helianthus debilis* var *cucumerifolious* and *Helianthus annuus* ssp *annuus* (Kim and Rieseberg 1999; Whitney et al. 2010), such transfer was hypothesized to account for the expansion of *Helianthus annuus* into novel environments and in producing its unique phenotype. These hypotheses were tested using a variety of experimental hybrid and parental genotypes/phenotypes, in both greenhouse and field experiments. A pattern of overrepresentation of *H. debilis* alleles suggested that these genomic components might confer a fitness advantage to the introgressed *H. annuus*. Additionally, introgression apparently altered multiple traits of the *H. annuus* phenotype causing adaptive changes in components interacting with the biotic and abiotic environments (Whitney et al. 2010).

The present study demonstrates species-level differences in connectivity among populations of Louisiana irises. In particular, *I. brevicaulis* populations appear much more isolated from intraspecific gene flow than do *I. fulva* populations. However, neither species demonstrates phylogeographic breaks indicative of the Mississippi River acting as a barrier to gene flow. In contrast to isolation among *I. brevicaulis* populations, we detected asymmetric introgressive hybridization of alleles largely from *I. fulva* into *I. brevicaulis*, with some of the allele transfer possibly being adaptive. For example, the introgressed, Alabama population of *I. brevicaulis* was found in an *I. fulva*-like habitat, that is, in standing water. Martin et al. (Martin et al. 2006) documented adaptive trait introgression of “flood-tolerance” alleles from *I. fulva* into *I. brevicaulis* that allowed the latter to survive an extreme flooding event. Performing a transplant study, in order to evaluate whether *I. fulva* parents or hybrid individuals survive as well or better in the *I. brevicaulis* collection site in Alabama, should provide a further test of the hypothesis of adaptive trait introgression within this species complex.

## Data Accessibility

The associated SNP genotypes for both within and between species (Hapmap format) along with GPS coordinates of collection localities are deposited in the Dryad data repository: doi: 10.5061/dryad.sd845.

## Supporting Information

Figure S1 Plots of posterior probabilities of group assignments of each individual into four clusters based on the STRUCTURE analysis for *Iris fulva*. The results are grouped by collection localities for each individual.

Figure S2 Inference of the number of genetic clusters by discriminant analysis of principle components (DAPC). The lowest Bayesian information criterion (BIC) value is found for (A) *Iris brevicaulis* to be 3 clusters and (B) for *Iris fulva* to be one cluster.

Figure S3 RAxML tree inferred using 387 concatenated SNPs showing the phylogenetic relationship of *Iris brevicaulis* populations. Individuals from Louisiana and Alabama form one clade, while all other individuals form a clade based on the respective collection locality.

Figure S4 RAxML tree inferred using 468 concatenated SNPs showing the phylogenetic relationship of *Iris brevicaulis* and *Iris fulva* populations.

Table S1 Observed and total heterozygosity and inbreeding coefficients as calculated by Genodive for all populations.
