# Supplementary figures and images for: Determining population structure and hybridization for two iris species
Source: Ecol Evol. 2014 Feb 17;4(6):743–55. doi: 10.1002/ece3.964 (PMC3967900; doi:10.1002/ece3.964)

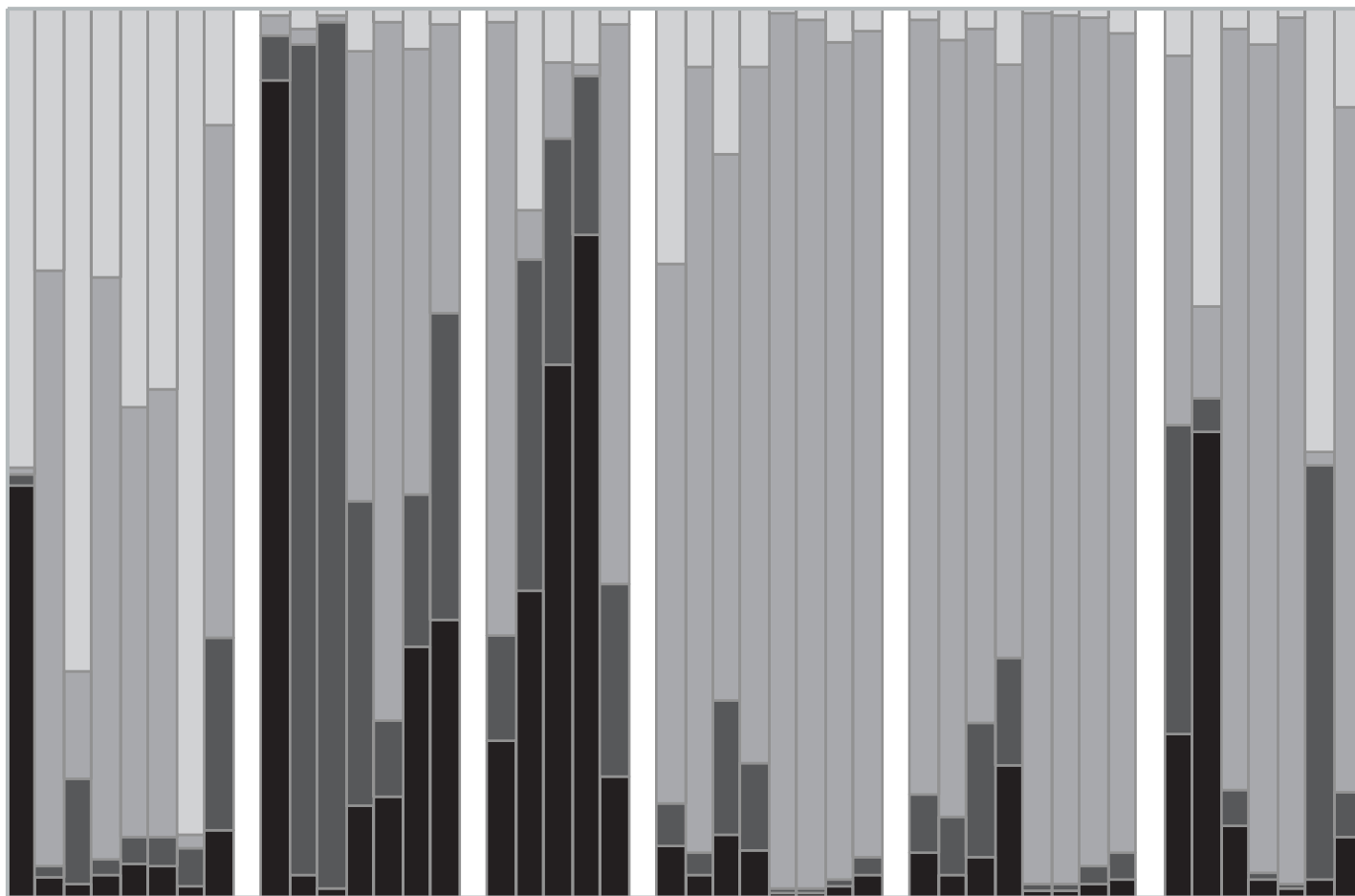

Arkansas

Illinois

Kentucky

Louisiana

Missouri

Mississippi

Supplement: Figure S1 — Plots of posterior probabilities of group assignments of each individual into four clusters based on the STRUCTURE analysis for Iris fulva. The results are grouped by collection localities for each individual. [file ece30004-0743-sd1.pdf]

a

Value of BIC  
versus number of clusters

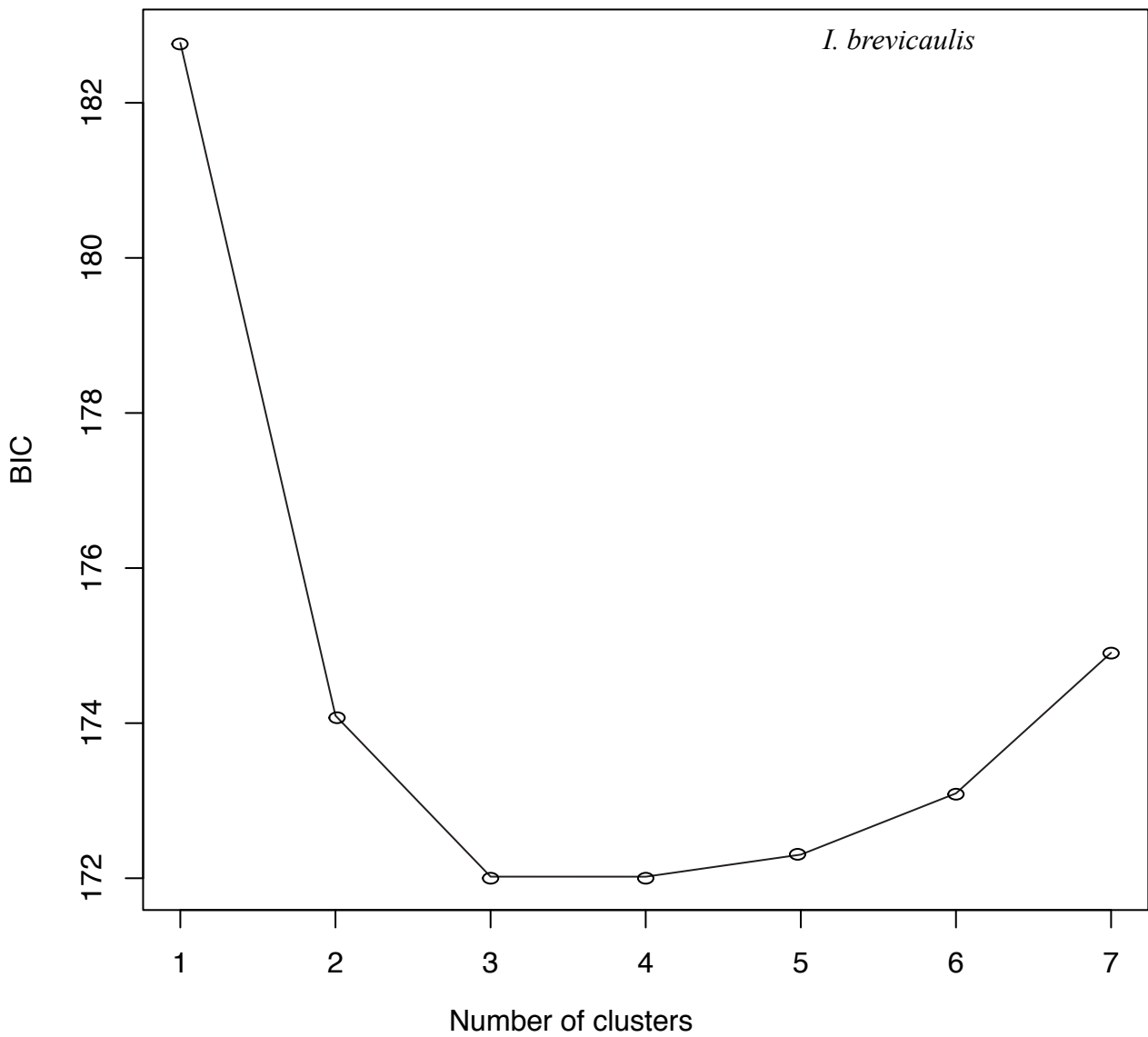

b

Value of BIC  
versus number of clusters

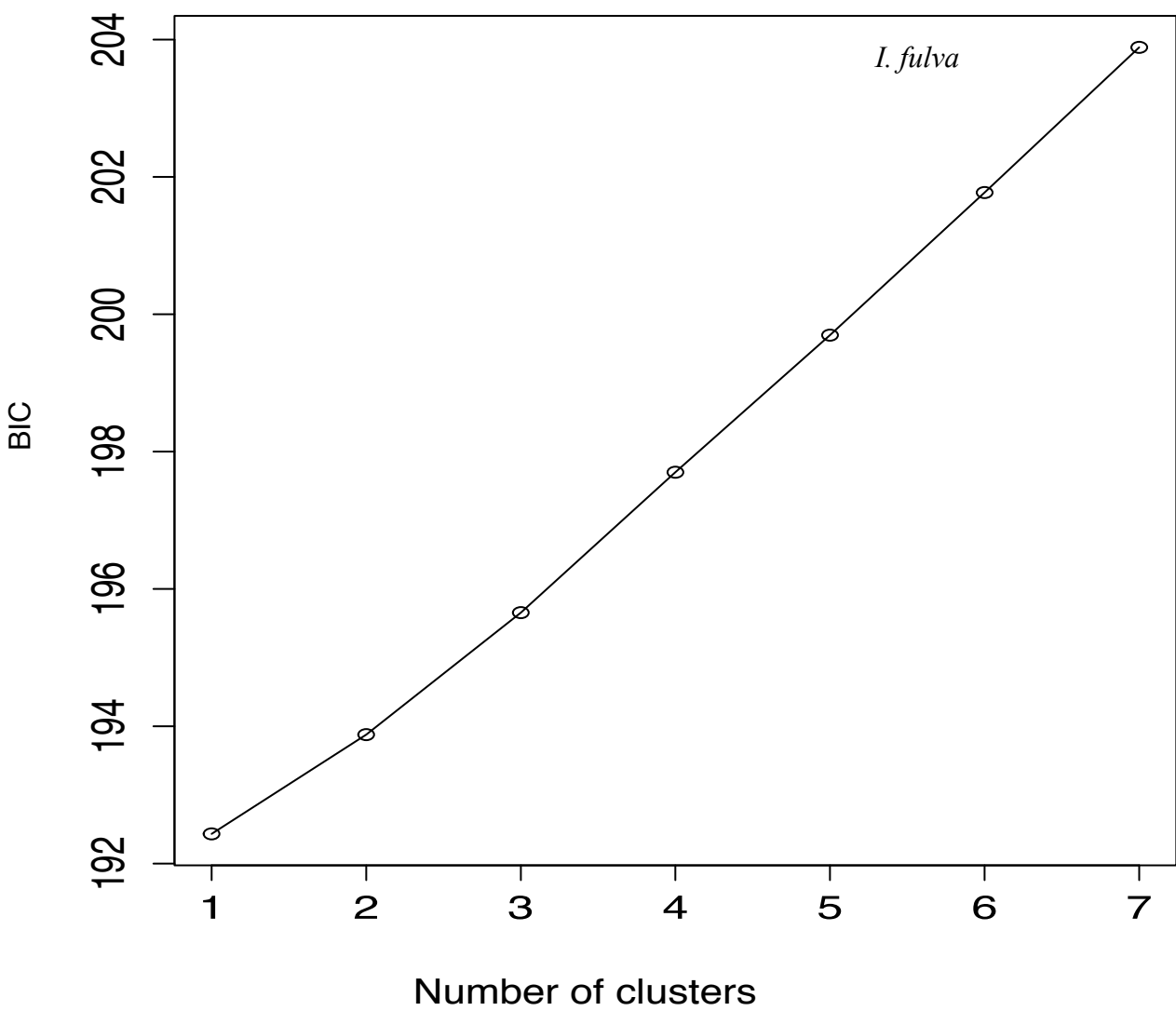

Supplement: Figure S2 — Inference of the number of genetic clusters by discriminant analysis of principle components (DAPC). The lowest Bayesian information criterion (BIC) value is found for (A) Iris brevicaulis to be 3 clusters and (B) for Iris fulva to be one cluster. [file ece30004-0743-sd2.pdf]

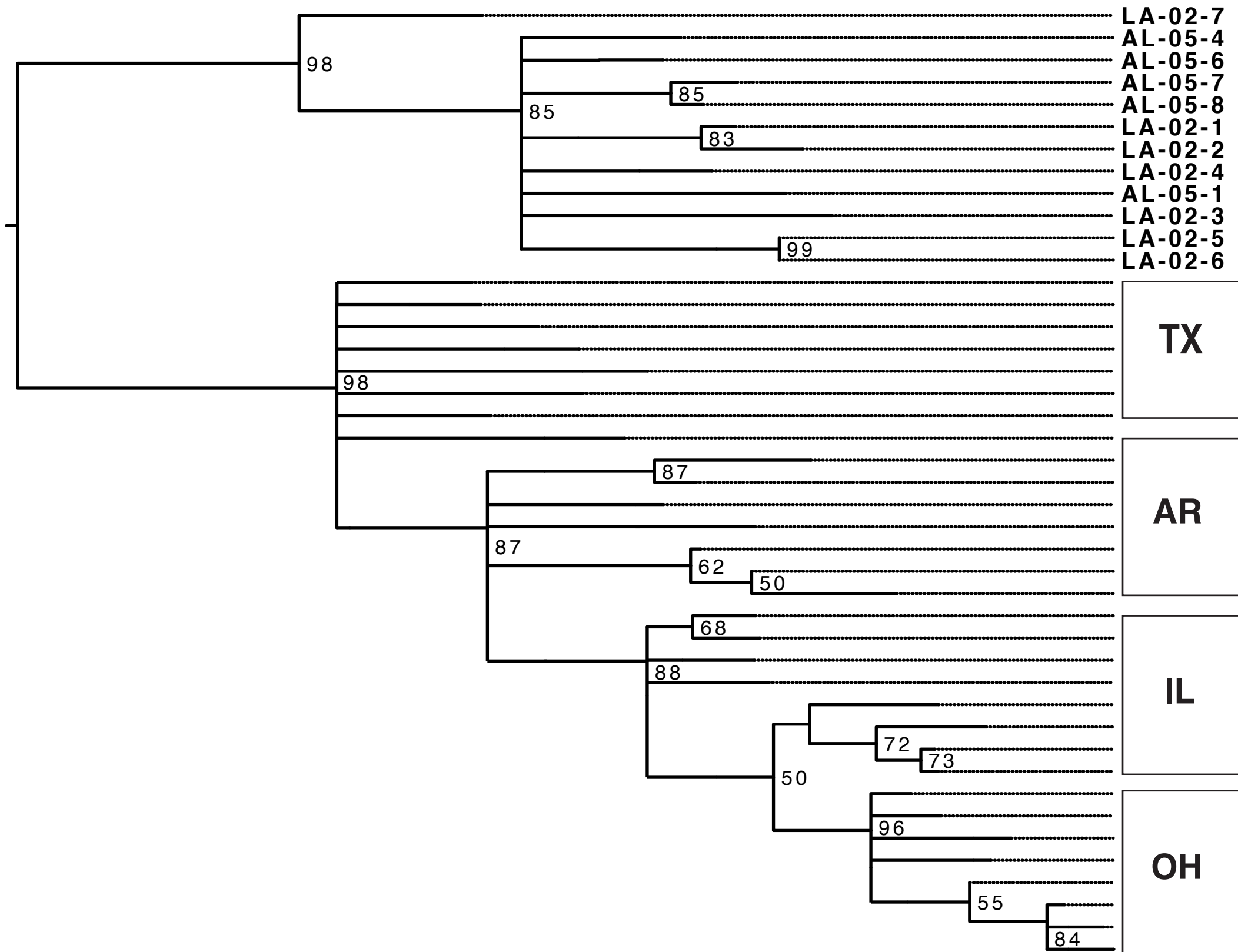

Supplement: Figure S3 — RAxML tree inferred using 387 concatenated SNPs showing the phylogenetic relationship of Iris brevicaulis populations. Individuals from Louisiana and Alabama form one clade, while all other individuals form a clade based on the respective collection locality. [file ece30004-0743-sd3.pdf]

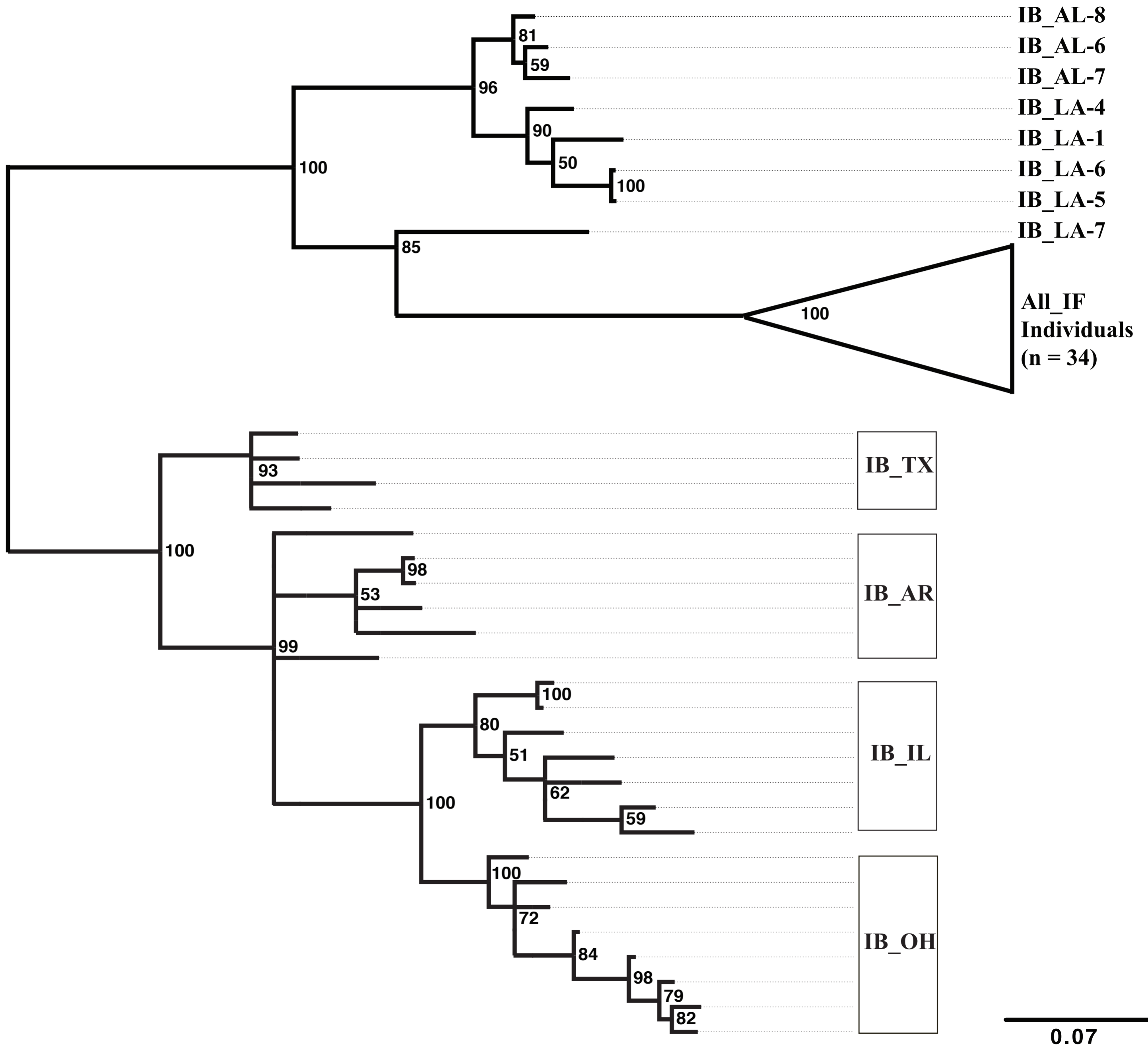

Supplement: Figure S4 — RAxML tree inferred using 468 concatenated SNPs showing the phylogenetic relationship of Iris brevicaulis and Iris fulva populations. [file ece30004-0743-sd4.pdf]
