# Supplementary material for: Determining population structure and hybridization for two iris species
Source: Ecol Evol. 2014 Feb 17;4(6):743–55. doi: 10.1002/ece3.964 (PMC3967900; doi:10.1002/ece3.964)
Supplement: Table S1 — Observed and total heterozygosity and inbreeding coefficients as calculated by Genodive for all populations. [file ece30004-0743-sd5.docx]

Supporting Table 1. Observed and total heterozygosity and inbreeding coefficients as calculated by Genodive for all populations. a) *I. brevicaulis* with 389 SNP markers and b) *I. fulva* 561 SNP markers. G_is_ values in bold are significantly different than zero.

| 1a | H_o_ | H_t_ | G_is_ |
| --- | --- | --- | --- |
| IB_AL | 0.101 | 0.144 | **0.3** |
| IB_AR | 0.212 | 0.217 | **0.024** |
| IB_IL | 0.172 | 0.189 | 0.09 |
| IB_LA | 0.156 | 0.19 | **0.175** |
| IB_OH | 0.259 | 0.201 | -0.291 |
| IB_TX | 0.104 | 0.145 | **0.282** |

| 1b | H_o_ | H_t_ | G_is_ |
| --- | --- | --- | --- |
| IF_AR | 0.38 | 0.357 | -0.066 |
| IF_IL | 0.328 | 0.361 | 0.09 |
| IF_KY | 0.314 | 0.347 | 0.097 |
| IF_LA | 0.545 | 0.403 | -0.351 |
| IF_MS | 0.561 | 0.399 | -0.405 |
| IF_MO | 0.447 | 0.384 | -0.164 |

H_o_ = Observed Heterozygosity, H_t_ = Total Heterozygosity, and G_is_ = Inbreeding coefficient
