# Supplementary material for: Determining population structure and hybridization for two iris species
Source: Ecol Evol. 2014 Feb 17;4(6):743–55. doi: 10.1002/ece3.964 (PMC3967900; doi:10.1002/ece3.964)
Supplement: Supplementary file 6 [file ece30004-0743-sd6.docx]

Supporting Figure 1. Plots of posterior probabilities of group assignments of each individual into four clusters based on the STRUCTURE analysis for *Iris fulva*. The results are grouped by collection localities for each individual.

Supporting Figure 2. Inference of the number of genetic clusters by discriminant analysis of principle components (DAPC). The lowest Bayesian information criterion (BIC) value are found for a) *I. brevicaulis* to be 3 clusters and b) for *I. fulva* to be one cluster.

Supporting Figure 3. RAxML tree inferred using 387 concatenated SNPs showing the phylogenetic relationship of *I. brevicaulis* populations. Individuals from Louisiana and Alabama form one clade, while all other individuals form a clade based on the respective collection locality.

Supporting Figure 4. RAxML tree inferred using 468 concatenated SNPs showing the phylogenetic relationship of *I. brevicaulis* and *I. fulva* populations.
